# Adaptive Mechanisms of Health Zones to Chronic Traumatic Events in Eastern DRC: A Multiple Case Study

**DOI:** 10.34172/ijhpm.2023.8001

**Published:** 2023-10-17

**Authors:** Samuel Makali Lwamushi, Hermès karemere, Robert Banywesize, Christian Molima Eboma, Pacifique Mwene-Batu, Corneille Lembebu, Giovanfrancesco Ferrari, Elisabeth Paul, Ghislain Bisimwa Balaluka, Philippe Donnen

**Affiliations:** ^1^Ecole Régionale de Santé Publique, Faculté de Médecine, Université Catholique de Bukavu, Bukavu, Democratic Republic of the Congo.; ^2^Ecole de Santé Publique, Université Libre de Bruxelles, Brussels, Belgium.; ^3^Hôpital Provincial Général de Référence de Bukavu, Bukavu, Democratic Republic of the Congo.; ^4^Division Provinciale de la santé du Sud-Kivu, Bukavu, Democratic Republic of the Congo.; ^5^Swiss Tropical and Public Health Institute, University of Basel, Basel, Switzerland.

**Keywords:** Adaptive Mechanisms, Health Zones, Traumatic Events, Eastern DRCongo

## Abstract

**Background:** The Eastern part of the Democratic Republic of Congo (DRC) has been affected by armed conflict for several years. Despite the growing interest in the impact of these conflicts on health service utilisation, few studies have addressed the coping mechanisms of the health system. The purpose of this study is to describe the traumatic events and coping mechanisms used by the health zones (HZs) in conflict settings to maintain good performance.

**Methods:** This multiple case study took place from July to October 2022 in four HZs in the South Kivu Province of DRC. HZs were classified into "cases" according to their conflict profile: accessible and stable (Case 1), accessible but remote (Case 2), unstable (Case 3), and intermediate (Case 4). Eight performance indicators and the amount of funding provided to the HZs by non-governmental organizations (NGOs) were recorded. A graph was created to compare their evolution from 2013 to 2018. A thematic analysis of qualitative data from individual interviews with selected health workers was conducted.

**Results:** Both battle-related events (war and its effects) and non-battle-related events (epidemics, disasters, strikes) were recorded according to the case conflict-profile. Although the cases (3 and 4) most affected by armed conflicts occasionally performed better than the stable ones (1 and 2), their operational action plan was poorly carried out. The coping mechanisms developed in cases 3 and 4 were the deployment of military nurses in preventive and supervisory activities, the solicitations of subsidies from NGOs, the relocation of health care facilities and the implementation of negotiation strategies with the belligerents.

**Conclusion:** Armed conflict results in traumatic events that disrupt the execution of the operational action plan of HZs. The HZs’ management team expertise, its strong leadership, and substantial financial support would enable this system to develop reliable and sustainable adaptive mechanisms.

## Background

**Key Messages**

**Implications for policy makers**

- Health zones (HZs) remained effective in terms of providing healthcare during armed conflict periods thanks to certain adaptive mechanisms put in place by the central office of these HZ: (1) use of military nurses in preventive and supervisory activities, (2) solicitations of non-governmental organisations (NGOs) subsidies, (3) relocation of some health facilities or creation of community care sites, and (4) negotiations with belligerents.
- Health system strengthening in armed conflicts-affected regions must focus on improving the leadership of the central office of the HZ and provide him with needful resources.
- Assessing the performance of armed conflict-affected HZ must take account of the adaptations implemented by healthcare professionals during armed conflict periods.

**Implications for the public**

This research shows that health zones (HZs) affected by armed conflict in Eastern Democratic Republic of Congo (DRC) have managed to maintain good performance in terms of health outcome indicators. This was achieved through the adoption of a number of mechanisms by the HZ management team during periods of conflict, augmented by financial support from non-governmental organisations (NGOs). This suggests that in order to support the population affected by the atrocities of armed conflict in Eastern DRC, the Congolese government should allocate a substantial proportion of its financial and technical support to these HZs. This would enable to provide health workers with the resources they need to remain effective and committed to providing quality health services to the population.

For more than two decades the eastern region of the Democratic Republic of Congo (DRC) has experienced violent armed conflicts.^1,2^ In 2020, there were 120 active armed groups in the region.^3^ Power disputes, interethnic conflict, territorial conquest, and the control of natural resource sites are among the multifaceted causes of these conflict.^4-7^ The consequences are a protracted crisis with both direct (deaths, abductions, property damage, and population relocation)^8-11^ and indirect effects. Several studies demonstrate indirect effects on the population’s well-being and education, psychological affects such post-traumatic stress disorder, and economic repercussions.^11–14^

 The health sector is among the most severely impacted by this chronic crisis. The demand for healthcare is affected by poverty and, above all, by the continuous displacement of the population exposing the population to acute life-threatening diseases (diarrhoea, malnutrition, cholera, and measles outbreaks).^15^ On the other side, attacks on medical workers, their kidnapping, and the destruction of medical facilities have a detrimental effect on the performance of the health system in terms of healthcare provision for the population.^16-19^ The measuring of performance, a multidimensional concept,^20^ is frequently done in low-income countries using only one dimension, goal accomplishment, without exploring other dimensions like coping or value maintenance.

 To deal with the shocks of armed conflict, coping mechanisms have been put in place, often with the assistance of international organisations. These mechanisms include de-escalation training for health workers to deal with attacks,^21^ the World Health Organization (WHO) initiative to monitor attacks on the health system in order to adapt humanitarian actions^22^ and the International Committee of the Red Cross platform on violence against health workers.^23^ Non-governmental organisations (NGOs) have taken on other components, such as the reconstruction of schools destroyed by conflict and the protection and social reintegration of civilians.^24,25^

 The Congolese health system is organised into three levels: the national normative level, the intermediate level, which serves as a supervisory level in each of the country’s 26 provinces, and the operational level, which is represented by the health zone (HZ) or health district.^26^

 Armed conflicts, mainly concentrated in the Eastern part of DRC, has a negative impact on population well-being,^27^ causes a high number of deaths^28^ and other adverse effects such as sexual violences.^29,30^ Although this is a major issue in eastern DRC, few studies have assessed the impact that armed conflict can have on the health system. The available literature focuses primarily on healthcare workers and facilities attacks,^31,32^ or the effect on services utilisation.^33,34^ The few studies in South Kivu that show certain mechanisms of adaptation of the health system to changing contexts (mobilisation of resources, rationalisation of health centre management, application of new standards, and dynamic community participation) in order to provide appropriate care to the population were conducted in HZs that had experienced little armed conflict since 2013.^35,36^

 The aim of this study is twofold: firstly, to characterise the traumatic factors that interfered with the provision of care, and secondly, to determine which coping mechanisms were put in place by the HZs to maintain an acceptable level of performance (according to the regulations in force in the DRC during the period of armed conflict).

## Methods

###  Study Design and Period

 This is a multiple case study. The reason for choosing this particular design is that it enabled us to examine how various HZs, that were adversely affected by armed conflict were able to adapt in order to continue providing care to the population.^37^ By comparing the different cases, it was possible to better understand why and how the HZs implemented these adaptation mechanisms to remain effective.^37^ The study was conducted from July to October 2022. The raw quantitative data collected ranged from 2013 to 2018.

###  Study Settings, Cases Selection, and Description

 The study was conducted in the eastern DRC province of South Kivu. In 2020, it had a population of 6 565 000, the majority of whom resided in rural areas.^38^ The operational level of the health system, the HZ, is subdivided into health areas with a geographical radius of 8000 to 15 000 km. Each of these health areas is covered by a health center and other optional health structures such as reference health centers and health posts. The health center (together with the optional structures) constitutes the first line of care in the HZ. The second line is the general referral hospital of the HZ. Especially in urban HZ, there may be optional secondary referral facilities (such as medical centers and clinics) and tertiary referral facilities (such as provincial and university hospitals).^39^ Each HZ develops an operational action plan, which outlines the critical activities that must be completed over the course of the coming year.

**Figure 1:**
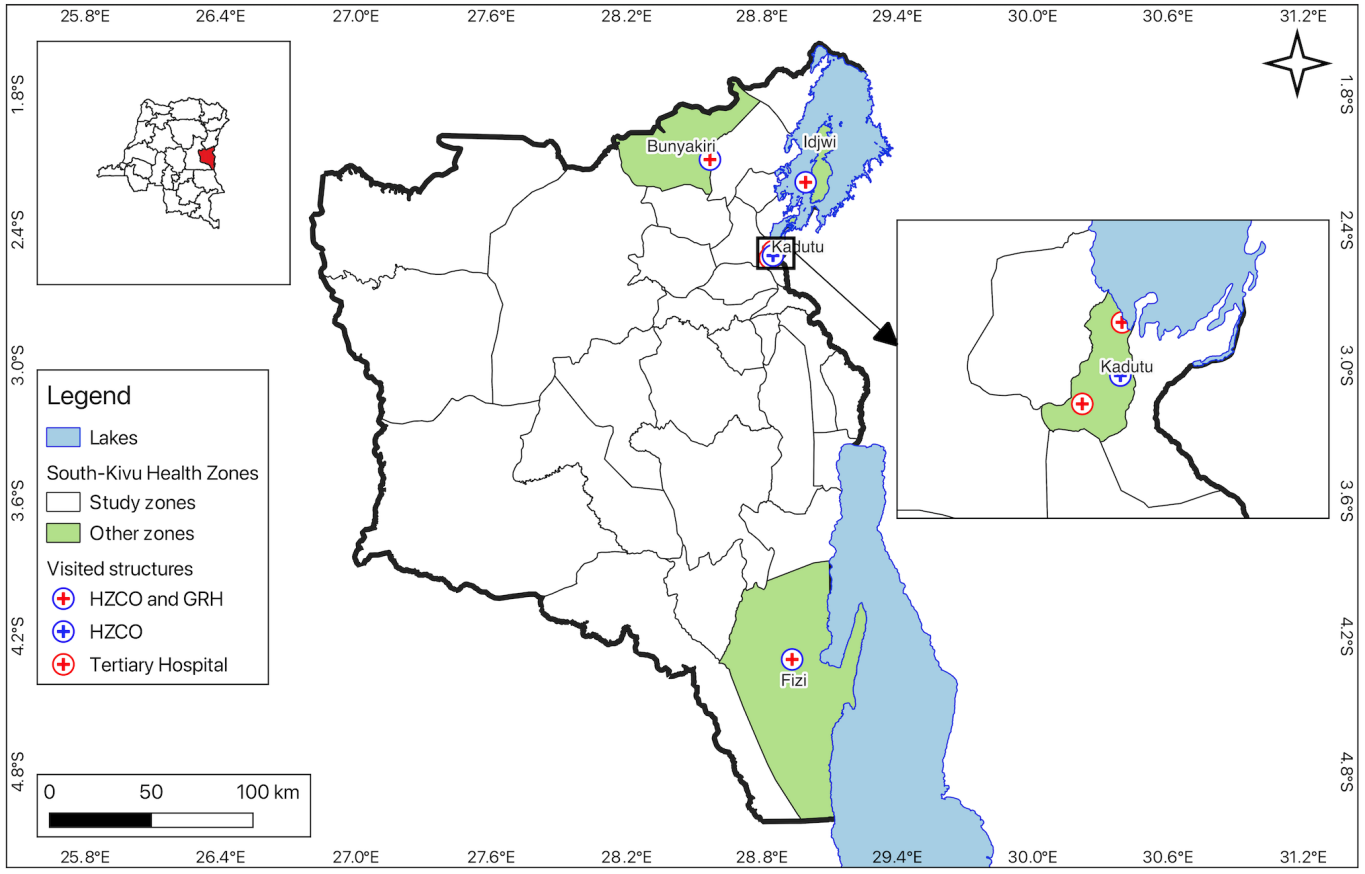
Selected Health Zones (source: performed by QIGS 3.30.1 based on Humaritarian Data Exchange shapefiles, provided by UNOCHA). Abbreviations: HZCO, health zone central office; GRH, General referral hospital; Tertiary hospilas, Hôpital Général de Référence de Bukavu and Ciriri hospital. Note: Some HZCO are located in the same place as the GRH (Fizi, Bunyakiri, and Idjwi health zones).

 We made the decision to use the HZ as a “case” because it serves as the functional component of the Congolese healthcare system. In order to provide a good opportunity to comprehend the adaptation mechanisms put in place, the selection criteria were based on the idea that the HZs should be diversified according to the degree of damage caused by armed conflict. To achieve this, 4 of the 34 HZs in the South Kivu province were selected based on the typology developed in a prior study^27^ (Figure 1). In order to exclude the periods of the two Kivu major wars (1996-2003) and those during which there were major rebellions in South-Kivu, we have chosen the period from 2013 (integration of the most influential armed group (Yakutumba) into the Congolese army)^6^ to 2018 (election of a new political regime to the head of the county).

 This study used 2 criteria to determine the level of exposure to armed conflict between 2013 and 2018: (*a*) the number of deaths directly related to armed conflict (battle-related death, BRD) using data from the Uppsala Conflict Data Program BRD Dataset, and (*b*) the number of internally displaced persons (IDPs) from the United Nations Office for the Coordination of Humanitarian Affairs (UNOCHA) database. The HZs that had ≥50 000 IDPs and ≥5 BRD/100 000 population were considered unstable, those with ≥50 000 IDPs and <5 BRD/100 000 population were considered intermediate and those with <50 000 IDPs and <5 BRD/100 000 population were considered stable. The classification of stable HZs also took accessibility into account.

 The first case was the urban HZ of Kadutu, easily accessible by land, which was considered as “stable and accessible.” There is an anarchic emergence of health facilities in this HZ that offer a poorly defined care package that is not integrated into the health system. The HZ has not experienced any major armed conflict since 2013. This HZ was chosen on purpose from the 3 urban HZs (which have the same stability and geographic accessibility characteristics) in South Kivu province in accordance with our previous study.^27^ The second case was the HZ of Idjwi, a rural island located 50 km from the shore of the city of Bukavu and accessible only by sea. Since the armed conflict in South Kivu began, this HZ has been spared because its difficult accessibility, which makes it landlocked compared to the rest of the HZs. So, it was considered as “stable and landlocked.” The third case was the HZ of Fizi, which is rural, can be accessed by road and sea, and had the highest number of BRDs and IDPs from 2013 to 2018. It was thus considered ‘unstable.’ The fourth case was the HZ of Bunyakiri, rural, accessible by road, which had many IDPs but few BRDs, and was considered as ‘intermediate.’ Rebels on the road hamper accessibility to this HZ (Table 1).

**Table 1:** Main Comparative Characteristics of Selected Cases

**HZ**	**Location/Accessibility**	**Administrative Territory/** **Number of Health areas**^d^	**Population (2019)**	**IDPs**^a^	**BRD**^b^	**Conflict Score**^c^	**Case Name**
Kadutu	Urban/by road only	Bukavu/13	380 501	0	0	0	Case 1: Accessible and stable
Idjwi	Rural/by sea only	Idjwi/21	294 209	0	0	0	Case 2: Accessible and remote
Fizi	Rural/by road and sea	Fizi/31	423 363	1	1	2	Case 3: Unstable
Bunyakiri	Rural/by road only	Kalehe/26	265 955	1	0	1	Case 4: Intermediate

Abbreviations: HZ, health zone; IDPs, internally displaced persons; BRD, battle-related death. ^a^Number of Internal displacement people: <50 000 = 0 and ≥50 000 = 1. ^b^BRD: <5/1 000 000 inhabitants = 0 and ≥5/1 000 000 inhabitants = 1. ^c^Score obtained by the health zones concerning the two criteria: 0 = stable, 1 = Intermediate, and 2 = unstable. ^d^According to the “Pyramide sanitaire du Sud-Kivu, 2019.”

###  Data Sources and Extraction

 To collect data, we combined both qualitative and quantitative methods. The qualitative approach allowed us to characterise the traumatic events that affected the HZs between 2013 and 2018 as well as the coping mechanisms put in place by these HZs. To collect this qualitative data, we used two types of methods: first, a review of the reports from the HZs available the provincial health division and from the NGOs that intervened in these HZs from 2013 to 2018. This review allowed us to identify some traumatic events and coping mechanisms for these events. After a quick analysis of the data from the reports review, we developed a guide to conduct subsequent semi-structured individual interviews to deepen the information from the review. The interview guide consisted of three main questions: (*i*) what major traumatic events occurred in your HZ between 2013 and 2018? (*ii*) which mechanisms did you put in place to deal with these events? and (*iii*) how did you proceed to implement these mechanisms?

 The interviews targeted healthcare workers who had lived in HZs that had recorded traumatic events related to armed conflict (cases 3 and 4) from 2013 to 2018 and who had agreed to participate in our study. Interviewees were selected on the basis of their ability to answer the questions outlined in the interview guide. Using the snowball method, the first respondents (mainly those from the central office of the HZ) guided us to other healthcare worker in the HZ who might have additional information. The choice of this second sampling approach was justified by the delicate nature of the questions asked. The interviews were conducted by the first author (SML) in French or Swahili, in a room within the health facilities (hospital, health centre, and HZ office) and lasted 30 minutes on average. A total of 13 health staff were interviewed (7 in case 3 and 6 in case 4) including medical directors, nurse attendants and supervisors, midwives, the director of nursing at the general referral hospital, the general administrator of the HZ, and other support staff. A dictaphone was used to record the interviews, and the audio files were saved on a computer.

 The quantitative approach consisted of describing the level of performance attained by each case from 2013 to 2018 using health indicators. Due to the difficulty of collecting the data required to carry out a multidimensional measure of performance, we used a unidimensional measure of goal attainment.^20^ The goal was considered to be the standard (value set by the Ministry of Health) that had to be achieved in order to be considered “successful.”^40^ To do this, we used routine health indicators collected at the HZ level. These paper-based data have been encoded in the national health information system (SNIS)^41^. Nevertheless, several data before 2017 were missing from this system. Using a pre-designed matrix, we collected additional data from the manually completed SNIS reports as well as from the annual reports archived at the central HZ office. Following consultation with the provincial health division experts and an assessment of the potential for indicators to be affected by armed conflict,^16-19,33^ eight indicators were selected to measure the performance of the different cases, as well as the amount of NGO subsidies to the HZ (Table 2). These indicators addressed the three levels of the health system in the HZ: first level (health centre), second level (general referral hospital) and third level (HZ central office). Only the data on the amount of NGOs subsidies to the HZ for case 1 in 2017 could not be found.

**Table 2:** Significance, Standard and Relationship With Armed Conflicts of Selected Health Indicators

**Health Indicator**	**Level of Health System**^a^	**Significance**^b^	**Relationship With Armed Conflict **	**Standard**^b^
Utilization rate of prenatal services 1 (ANC1)	1st	Reflects acceptance of the service and recruitment of pregnant women	During armed conflicts, preventive activities are often abandoned in favour of curative health emergencies	80%-100%
CSR	1st	Measures the extent to which people use the service	Accessibility to health services is often limited in areas affected by armed conflict	60%-100%
DR3^c^	1st	Appreciates the accessibility of the vaccination service by children	During armed conflicts, preventive activities are often abandoned in favour of curative health emergencies	0%-10%
ADR	1st and 2nd	Appreciates the management of deliveries by a trained person	Delivery in appropriate conditions may be affected by armed conflict	80%-100%
BOR	2nd	Evaluates the use of the hospital, assesses the quality of services	Hospitals can sometimes be crowded with seriously injured or ill patients during armed conflicts	80%-100%
IHR	2nd	Capacity for adequate inpatient care	Mortality is increased in hospital during armed conflicts, often related to serious bullet wounds	<2%
Proportion of action PAC	3rd	Appreciates the ability to monitor the level of achievement of the objectives set out in the action plan	The previously defined action plan is often forgotten in order to deal with health emergencies	80%-100%
Proportion of supervisions performed (SUP)	3rd	Enables staff to be monitored, training needs to be identified, staff performance to be improved and service quality	Supervision of health facilities in conflict affected areas may be compromised	80%-100%
ANGO	2nd and 3rd	Reflects the level of contribution of NGOs to the financial accessibility of care	In armed conflict settings, humanitarian aid is often extensive, managing health emergencies	**-**

Abbreviations: IHR, in-hospital mortality ratio; CSR, curative service utilisation rate; DR3, drop-out rate for the 3rd dose of pentavalent vaccine; ADR, assisted delivery rate; BOR, bed occupancy rate; NGOs, non-governmental organisations; PAC, plan activities completed; ANGO, Amount of funding received from NGOs; ANC1, utilization rate of prenatal services 1; SUP, proportion of supervisions performed. ^a^Level of health system in the health zone: 1st = health center, 2nd = general referral hospital, and 3rd = Health zone central office. ^b^Significances and standards are extracted from the DRC Ministry of Health catalogue of indicators (October 2004) and national health development plan (2019-2022). ^c^Pentavalent vaccine is a combination of 5 vaccines: diphtheria, pertussis, tetanus, hepatitis B, and Haemophilus influenzae type B.

###  Data Analysis

 The quantitative data were coded and analysed using Microsoft Excel 2016. Trend analysis of key health performance indicators for the different cases was performed. The difference in standards attainment for these indicators for each case was plotted from 2013 to 2018.

 For the qualitative data, the audio files (in French and Swahili) from the interviews were transcribed into Word files (in French). We then carried out a thematic analysis^42^ to describe the experiences (traumatic events and coping mechanisms) of health workers in HZs affected by armed conflict. The first phase consisted of familiarisation with the data. Then, a manual analysis of the transcripts was carried out by two persons separately (SML and CME) to establish codes. These different codes were grouped into two main themes: (*a*) the traumatic events experienced by the health staff from 2013 to 2018; and (*b*) the coping mechanisms implemented by these staff to maintain a good performance of the HZ. Regarding traumatic events, a timeline of traumatic events was developed from 2013 to 2018. To ensure maximum confidentiality in quoting the verbatims, only the interviewees’ title (nurse, physician, administrative, midwife, and support staff) and the type of HZ (case 3, case 4) were retained.

 Finally, a cross-case analysis^37^ was carried out to link the context, the traumatic events, the coping strategies and the level of performance in each case.

## Results

###  Qualitative Results

####  Main Traumatic Events in the Affected Health Zones From 2013 to 2018

**Figure 2:**
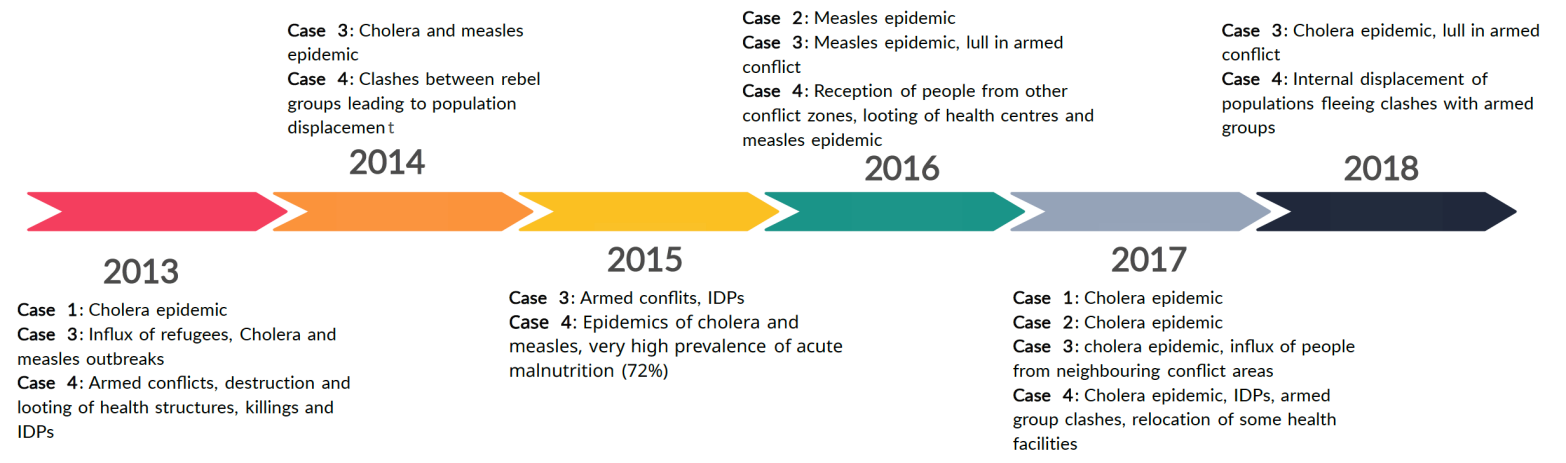
Main Traumatic Events in Case 3 (Unstable HZ) and Case 4 (Intermediate HZ) From 2013 to 2018. Abbreviations: HZ, health zone; IDPs, internally displaced persons.

Figure 2 shows the main traumatic events that occurred in the different HZs from 2013 to 2018. These events are classified into two groups: first, battle-related events (BREs), ie, armed conflicts and their consequences and non-battle related events (NBREs). The first group of events was not reported in the stable cases (cases 1 and 2) and refer to conflicts between armed groups. These were either battles between two ethnic groups in conflict, or between armed rebels and the Congolese army.

 The difference between these two types lied in the causes of confrontations. In the first case (inter-ethnic conflicts), the main cause was the possession of land and property (for example, the spoliation of the cattle of one group by another). In the second case (clash between armed rebels and Congolese army), the principal cause was the gain of power over a given territory. For both types of conflicts, there were direct consequences such as killings, rapes, property destruction (including health facilities) and internal displacement (people moving from neighbouring areas into already unstable HZs). Displaced persons mixed with the population of the HZ by settling with host families. The armed rebels could also settle along certain roads to loot, and even kidnap people.

 “*The main cause of these wars are inter-ethnic conflicts... In 2017, we can point to the conflict between herders and farmers, which took place in the middle plateau. The cows of the herders went to graze in the fields of the indigenous people of this area and there were clashes between these two peoples” *(Nurse, case 3).

 “*Since I have been in the health zone (13 years)... I have witnessed many atrocities and movements of the population (internal displacements)... these movements have affected the functioning of the health zone in the event that a health area moves to another area; go and understand by deduction of mass displacement, promiscuity, malnutrition and the enclosure of a health area where people have been emptied out...” *(Nurse, case 4).

 The second type, the NBRE, were occasionally observed in the stable cases (1 and 2). They consist of other events that have an impact on the functioning of the health system, including recurrent epidemics of cholera and measles as well as very high prevalences of malnutrition. It was also reported that health workers went on strike for long periods of time due to unpaid bonuses. In case 3, there were also recurring natural disasters (overflow of Lake Tanganyika destroying the road to the HZ).

 “*...These clashes are events that have marked our health zone. But in the meantime, we have the cholera epidemic which has become endemic. Recently we have also had a measles epidemic and what caused this measles epidemic was the nurses’ strike which lasted for 8 months to claim their salaries and bonuses...” *(Nurse, case 3).

 “*...the overflowing of Lake Tanganyika which caused destruction of houses and even roads. This negatively affected the health zone. There were difficulties of displacement between Fizi and Uvira ” *(Administrative, case 3).

####  Adaptive Mechanisms Implemented by Affected Health Zones 

 Interviewees and the various reports consulted both mentioned coping mechanisms for traumatic events. The first mechanism, essentially cited in Fizi (case 3), was the use of military nurses (from the DRC armed forces) to carry out preventive activities (eg, vaccination, distribution of mosquito nets) and supervision in some case 3’s health areas. The HZ management team provided these nurses with rapid training. Their remuneration was derived from the financial resources directly involved in the activities supported by the non-governmental partners. The HZs was sometimes forced to dip into its reserves to pay the military nurses. These mechanisms were created to provide care in a health area affected by armed conflict while the population was still present.

 “*To intervene in a health area in conflict, we have put in place a homemade strategy. We intervene through military collaboration. We contact the DRC military forces’s nurses and ask them to help us carry out supervisions and accompany mass activities such as vaccination, distribution of mosquito nets and other activities” *(Physician, case 3).

 Secondly, the grant from NGOs was the most frequently cited mechanism by the respondents. NGOs support was mor frequently cited in Fizi (case 3) than in Bunyakiri (case 4). These NGOs not only provided financial but also technical support to the HZ. The first support consisted of a lump sum given to the central office of the HZ or to the general referral hospital for their functioning, a lump sum bonus paid to agents who intervene in a specific programme, and the provision of medical equipment and inputs to the health facilities and central office of the HZ. The second type of support included strengthening the skills of the health staff through continuous training or practical accompaniment by technical assistants. The HZ’s core team assessed displaced communities’ health needs and used this information to advocate to attract NGO funding. Unfortunately, the rebels occasionally kidnapped some of the NGO workers, often resulting in the cessation of support to the HZ.

 “*They (displaced persons or IDP) have IDP badges; on these badges it is marked IMC (International Medical Corps). When they arrive at the hospital, they just show the badge, and they get free treatment” *(Midwife, base 3).

 “*It was on the road, at about 2 p.m …. the project manager of MSF (Médecins Sans Frontières ) and his entire team were kidnapped. This bad event was the precursor of the big unfortunate decision of MSF departure from the health zone. Alongside … was the total abortion of the Baraka hospital construction project” *(Physician, case 3).

 The third coping mechanism identified was the relocation of the health facilities. It was either the health staff who moved to open a community site in non-conflict areas where the population had sought refuge when fleeing the war (mainly in Fizi, case 3). In this first case, when the situation calms, the population returns to its natural environment and the community site is closed. Alternatively, if the conflict is permanent, the central office of the HZ may decide to relocate the health facility, which is also vulnerable to destruction during the clashes (mainly in Bunyakiri, case 4).

 “*To facilitate accessibility and to ensure that quality care reaches the entire population of the district, we created healthcare sites in the health areas. There were community health sites, and we also set up health posts for all the people who are more than 5 kilometres away and who had difficulty accessing the health centres” *(Nurse, case 3).

 “*...when the situation here in the health centre became critical at the time of the rebels, the general hospital moved…. What I can say about 2017 is that most of the health centres stopped functioning in their usual places and moved to other places...” *(Support staff, case 4).

 The last mechanism mentioned by the interviewees, both in Fizi (case 3) and Bunyakiri (case 4) was the implementation of negotiation strategies with the belligerents. This involved carrying out as many activities as possible during calm periods and limiting actions during times of armed conflict. Sometimes, health staff would negotiate with rebel leaders to keep them out of conflict and secure healthcare sites. Community leaders usually mediated these negotiations.

 “*The health zone is in a territory that has long been called the “red zone”; we are already used to it. Clashes can take place today and the day after tomorrow we continue our activities” *(Nurse, case 3).

###  Comparative Trends in Key Performance Indicators and Case Analysis

**Figure 3:**
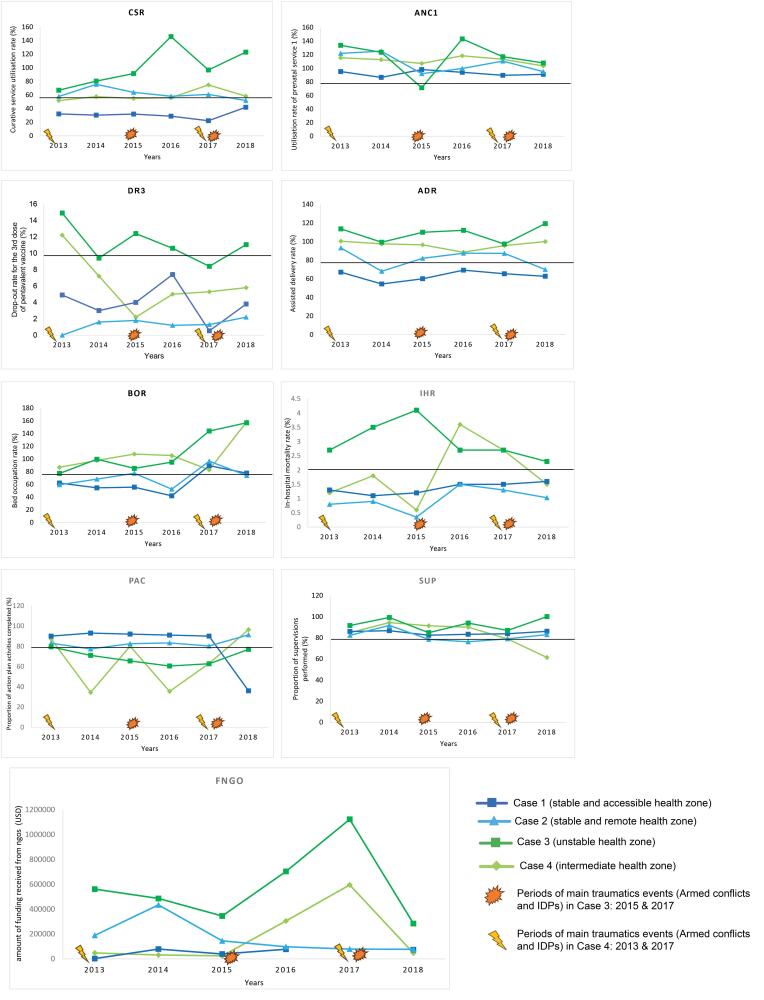
Trends in the Level of Achievement of Key Performance Indicators and NGO Subsidies in the 4 Cases From 2013 to 2018. Note: The black line on the trend’s charts indicates the norm for good performance in DRC for each indicator. Abbreviations: CSR, curative service utilisation rate; DR3, drop-out rate for the 3rd dose of pentavalent vaccine; ADR, assisted delivery rate; BOR, bed occupancy rate; IHR, in-hospital mortality ratio; NGO, non-governmental organisation; PAC, plan activities completed; ANC1, utilization rate of prenatal services 1; SUP, proportion of supervisions performed.

Figure 3 shows the evolution of trends in performance indicators as well as the amount of NGO funding allocated to the HZs.

 The stable and accessible HZ (case 1) received the least amount of NGO subsidies. Its context is quite complex, with the emergence of several secondary level health facilities, often for profit, suffocating the health centres, and the presence of two tertiary level hospitals managed by ministry of health (Hôpital Provincial Général de Référence de Bukavu and the Ciriri Hospital). Periodic NBREs have been observed in case 1. Certain performance indicators, on the other hand, are poor (service utilisation rate <45%, bed occupancy rate <60% from 2013 to 2016, and an assisted delivery rate ≤ 70%). Nonetheless, case 1 is the most efficient in carrying out the operational action plan’s activities (90%-93% execution rate), except in 2018 (36%), and supervisions (>80% execution rate). Preventive activities (prenatal consultation, pentavalent vaccination) and in-hospital mortality remain within the acceptable norms.

 Because of its location the stable and remote HZ (case 2) (island in Lake Kivu) is the only one that has not experienced armed conflict in South Kivu. Only NBRE were reported in this HZs. The majority of the performance indicators are favourable (in-hospital mortality, low pentavalent 3 vaccine drop-out rate, assisted deliveries, supervisions, and antenatal consultations). The operational action plan has been implemented to more than 80%. The two indicators that did not meet the standard in this case were the service utilisation rate (52%-75.7%) and the bed occupancy rate (<80% except for 2017). NGO grants have been low except in 2014, with a peak of over US$ 400 000. This year saw a paradoxical decrease in the implementation of operational action plan activities (77.3%), an increase in the use of care services (75.7%), and an increase in supervision (91.7%).

 For case 3 (unstable HZ), three situations emerged: first, there was a drop in performance indicators when the HZ had many BREs and the amount of funding received from NGOs (ANGO) were low (2015). Second, even when the ANGO was low but the HZ was facing NBREs (2013, 2014, and especially 2018), the performance indicators tended to improve. Third, when the ANGO was high, regardless of the type of traumatic events (2016 and especially 2017), the trend in performance indicators improved until they exceeded the normality thresholds. During this period only the proportion of action plan activities completed (PAC) remained the weakest indicator. According to the respondents, action mechanisms were in place in all three situations but more so when there were fewer BREs and financial support was high.

 For case 4 (intermediate HZ), even though the ANGO are low and the HZ is facing BREs (2013 to 2014), the performance indicators (except for PAC) were still within the norms. According to interviewees, the HZ is used to absorb the shock of the chronic crisis and to put in place coping mechanisms that are not reliant on NGO subsidies (negotiation strategies, relocation of care sites). In comparison to Case 3, this subsidy was very low during this period. However, as in case 3, when there were more subsidies, the level of achievement of the indicators improved despite the type of traumatic events (2016 and 2017).

## Discussion

 The objective of this study was to describe the traumatic events that occurred in HZs in chronic crisis due to armed conflict from 2013 to 2018, as well as the coping mechanisms put in place by these HZs to maintain good performance. Our findings indicate that the traumatic events that occurred during this time period that could have an impact on the health system BREs, consisting of clushes between armed groups and their direct consequences (deaths, kidnappings and relocation of health facilities), and NBREs, which included events such as epidemics, natural disasters and health personnel strikes. The HZs in affected by armed conflicts (cases 3 and 4) maintained an acceptable level of performance for some performance indicators but their level of PAC achievement remained low. The use of military nurses in the affected areas, the relocation of health facilities, attracting NGO funding and implementing negotiation strategies with the belligerents were among the coping mechanisms identified.

 The coping mechanisms put in place by the HZs in crisis depended on the traumatic events they experienced. The BREs were the events that had the greatest impact on the health system as they caused a drop in the performance of the indicators in these HZs. These results corroborate those of other studies that have shown the vulnerability of health systems during armed conflicts.^16,19,33,43^ The NBREs in crisis areas were additive factors to the BREs which could explain the destabilisation of the health system.^15^ In contrast, in stable HZs, these NBREs could be regarded as emergent and ephemeral events, leading to the rapid and effective implementation of appropriate measures. Maintaining good performance in this situation was dependent on the coping mechanisms put in place by the health workers, without external assistance. This would be explained by strong leadership and organisational governance within these HZs. However, with financial support from NGOs, the performance of health indicators improved. Indeed, the health system faces several challenges during armed conflicts, including the lack of appropriate support.^44^ In some African countries, such as the DRC, where resources allocated to the health sector remain below the 15% threshold set in the Abuja Declaration,^45^ the majority of funding for this sector comes from external partners.^26,46-48^ The government’s support is mainly focused on the payment of risk premiums to certain healthcare staff (mainly doctors and nurses).

 In the crisis HZs (cases 3 and 4), performance was better for certain indicators such as bed occupancy rates and use of curative services when compared to the stable HZs (cases 1 and 2). This could be explained by the urban health context,^49^ which is characterised not only by socio-economic degradation^50^ but also by the disordered emergence of health facilities that are not integrated into the health system and offering a poorly defined care package for case 1. Furthermore, this HZ has two tertiary level hospitals for which the health system has no data collection framework, even though these are the most frequented health facilities.^39^ The explanations for case 2 are, however different. The predominance of traditional medicine, difficult financial access to care, and the low technical level of the health facilities (which are underfunded) could all be explanatory factors for this situation. Also, the isolation of case 2 could be a major factor explaining the low level of these indicators.^51,52^

 Finally, three indicators seem to reflect the state of crisis in the affected HZs: high intra-hospital mortality and high rate of pentavalent 3 vaccine drop-out, as well as a low rate of operational action plan completion. This can be explained firstly by the fact that there were no BREs but sporadic NBREs in the stable HZs from 2013 to 2018, so the drop-out rate for pentavalent 3 vaccine and in-hospital mortality could be low. Subsequently, in the event of a crisis, the HZs prioritises resolving health emergencies (management of war casualties and epidemics) and establishing coping mechanisms to the detriment of activities planned in the operational action plan.

###  Study Strengths and Limitations 

 The strength of this study is that it shows how the different coping mechanisms developed in crisis-affected HZs can contribute to maintaining its performance even during periods of conflict. On the other hand, tracing traumatic events over a period of 6 years allowed us for a better understanding of trends in the achievement of different performance indicators in the affected HZs. This was made possible by using a combination of methods to triangulate information.

 Nevertheless, some study limitations should be highlighted. The memory bias that some health workers may have in recalling traumatic events and coping mechanisms. This bias was offset by questioning other sources, including the reports review. The absence of some of the 8 selected indicators due to the fact that they were not encoded in the SNIS before 2017 was remedied by collecting the raw data at the HZ level. These raw data collected within SNIS reports as well as from the HZ annual reports, had already been verified at the annual HZ meeting. To minimize the risk of error, we had these data validated by the expert from the provincial health division in charge of health information system (DIHS2).

 Knowledge of the health system in the Eastern DRC and experience of previous studies in areas of armed conflict could influence the authors’ position on the opinions of the health workers interviewed. To minimise this bias, we drew up the interview guide on the basis of a preliminary literature review, the questions asked were open-ended to allow people to express themselves as much as possible, and the data were analysed by two separate authors (SML who conducted the interviews and CME) so as not to modify what the interviewees had to say. Finally, the results of the thematic analysis were reviewed by other authors (EP and GF) who were less familiar with the crisis context linked to the armed conflicts in eastern DRC.

## Conclusion

 The chronic crisis caused by armed conflicts leads to traumatic events that affects the performance of the health system in South Kivu. In the face of this shock, health staff in the HZ have put in place coping mechanisms to continue providing healthcare to the population. These initiatives were facilitated in part by financial support from NGOs. In the event of chronic armed conflict, the HZ management team, with good leadership and organisational culture, and sufficient resources, could serve as the foundation for adapting this system. A study to define a resilience framework for the HZ in chronic armed conflict would be beneficial.

## Acknowledgements

 We sincerely thank the health provincial division of South Kivu for his contribution to this research.

## Ethical issues

 This study was approved by the ethics committee of the Catholic University of Bukavu (UCB/CIES/NC/017/022). Authorisation from the South Kivu provincial health division was obtained before data collection in the health zones. Verbal informed consent was systematically obtained from interviewees before the recording.

## Competing interests

 Authors declare that they have no competing interests.

## Disclaimer

 The views expressed in this article are author’s and not an official position of the institution or funder.
